# *Methylobacterium* as a key symbiont in plant-microbe interactions: Its ecological and agricultural significance

**DOI:** 10.5511/plantbiotechnology.25.0309a

**Published:** 2025-09-25

**Authors:** Cecilia Eugenia María Grossi, Rita María Ulloa, Nurettin Sahin, Akio Tani

**Affiliations:** 1 Laboratorio de Transducción de Señales en Plantas, Instituto de Investigaciones en Ingeniería Genética y Biología Molecular (INGEBI), Consejo Nacional de Investigaciones Científicas y Técnicas (CONICET), Ciudad Autónoma de Buenos Aires, Argentina; 2 Departamento de Química Biológica, Universidad de Buenos Aires (UBA), Ciudad Autónoma de Buenos Aires, Argentina; 3 Egitim Fakultesi, Mugla Sitki Kocman University, Mentese, Mugla, Turkiye; 4 Institute of Plant Science and Resources, Okayama University, Okayama 710-0046, Japan

**Keywords:** bioinoculant, phyllosphere, plant growth-promoting rhizobacteria (PGPR), plant-*Methylobacterium* interactions, rhizosphere

## Abstract

Pink-pigmented facultative methylotrophs (PPFMs), encompassing the genera *Methylobacterium* and *Methylorubrum*, can utilize reduced one-carbon compounds such as methanol, methylamine, formaldehyde, and formate as carbon and energy sources. They are commonly associated with plants, particularly on leaf surfaces (phyllosphere), where their methylotrophic metabolism offers a significant adaptive advantage over other bacterial species. These genera hold quite diverse species with unique phenotypes. Many studies report plant growth-promoting activity of the genera due to their ability to produce plant hormones and help plants acquire nutrients. Also, the ecology of the genera that enables them to survive in such a harsh environment exposed to ultraviolet light, fluctuating temperature and humidity, and limited nutrients is the key to understanding their diversity, functions, and adaptations supported by their genotypes. In this review, we summarize their taxonomy diversified by their genotypes and niches, functions involved in plant growth promotion and survival in the phyllosphere, and practical application of the bacteria for agricultural purposes.

## Introduction

To meet the global challenge of feeding humanity, food production must increase by 70% by 2050. As highlighted by the Food and Agriculture Organization of the United Nations (FAO; http://fao.org), the agrifood systems face important pressures due to climate change, land degradation, and biodiversity loss. These facts, combined with rapid population growth and urbanization, threatens our capacity to provide nutritious food sustainably. These complex, interconnected challenges demand a holistic, systems-based approach.

The Green Revolution of the mid-20th century dramatically transformed agricultural practices and boosted food production (Pingali 2012). This transformation introduced mechanization, high-yielding dwarf varieties of wheat and rice, and widespread use of fertilizers and pesticides. However, this agricultural paradigm became more dependent on chemical fertilization and genetically uniform crops. Moreover, the continuous use of agrochemicals has revealed serious detrimental effects on both agricultural ecosystems and human health.

In nature, plants are closely associated with a diverse array of microorganisms that colonize every part of their bodies. These plant-microbe interactions have evolved into intricate networks of cooperation and antagonism, shaping plant health and ecosystem dynamics. Among these microbial partners, bacteria play a paramount role in forming plant microbiomes that enhance crop resilience and contribute to ecological balance. The concept of utilizing plant growth-promoting rhizobacteria (PGPR) dates back to the late 19th century. These beneficial bacteria enhance plant growth through multiple mechanisms, including nitrogen fixation, phosphate solubilization, phytohormone production, and biocontrol of plant pathogens (Ehinmitan et al. 2024). In the 21st century, research into plant-microbe interactions has intensified, driven by the urgent need for sustainable agricultural solutions. Among PGPR, the genera *Methylobacterium* and *Methylorubrum* emerge as a significant part of plant microbiomes (Palberg et al. 2022). Their capacity to produce phytohormones (particularly cytokinins and auxins), combined with their methylotrophic ability and highly adapted plant-associated lifestyle, positions them as excellent candidates for developing next-generation biofertilizers and engineered synthetic microbial consortia.

## Taxonomy and phylogenetic position of *Methylobacterium* species

Pink-pigmented facultative methylotrophs (PPFMs), which currently comprise the genera *Methylobacterium* (Patt et al. 1976) and *Methylorubrum* (Green and Ardley 2018), are Gram-stain-negative bacteria that can synthesize carotenoids and grow on reduced one-carbon (C1) compounds such as methanol, methylamine, formaldehyde, and formate. They are strictly aerobic, rod-shaped organisms belonging to the class *Alphaproteobacteria*, order *Hyphomicrobiales* (synonym=*Rhizobiales*) (Hördt et al. 2020).

The first PPFM described in the literature was an oxalate-utilizing (oxalotrophic) bacterium (Bassalik 1913; Sahin et al. 2008) isolated from earthworm contents and initially named *Bacillus extorquens*. Later, this strain, along with other previously identified PPFMs, was reclassified under the genus *Protomonas*, with *P. extorquens* (type strain NCIMB 9399) designated as the type species (Urakami and Komagata 1984). Subsequently, the genus name was replaced with *Methylobacterium* due to nomenclatural priority rules, as the genus *Methylobacterium* had been proposed earlier by Patt et al. (1976) with *Methylobacterium organophilum* (strain XX=ATCC 27886) as the type species.

In 2018, a group of 11 *Methylobacterium* species was reclassified under a new genus, *Methylorubrum*, with *Mr. extorquens* designated as the type species, based on multi-locus sequence analysis, genome data, and phenotypic characteristics (Green and Ardley 2018). At the time of writing (Feb 2025), the genus *Methylobacterium* comprises 61 effectively published species, with five validation-pending, while the genus *Methylorubrum* contains 12 validly published species (https://lpsn.dsmz.de/). For consistency, all species names in this review have been updated to reflect their current taxonomic designations. Alessa et al. (2021) sequenced 29 type strains, completing the genomic data of the genus, and assessed their phylogenetic and phenotypic diversity. With other studies (Hördt et al. 2020; Leducq et al. 2022), doubts have been raised about the genetic distinctions used to separate *Methylorubrum* from *Methylobacterium*.

**Figure 1:**
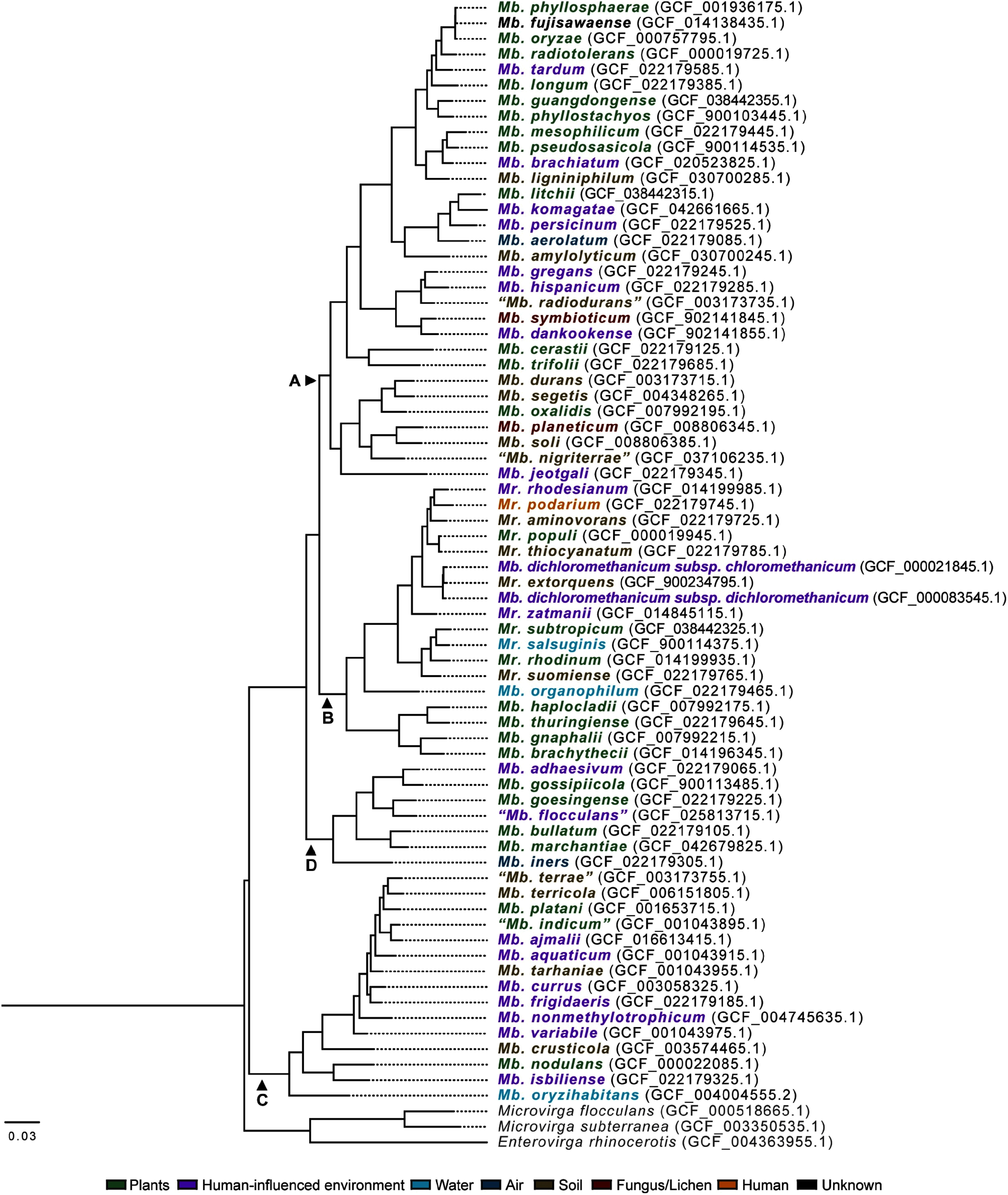
Figure 1. Methylobacterium/Methylorubrum whole-genome phylogeny based on 107 single-copy core genes. Maximum-likelihood analysis was inferred using the LG+F+I+G4 model. A total of 35,950 amino-acid positions were used. Phylogenetic groups A, B, C, and D are indicated with black triangles, while different colors represent the original isolation source of each species. The outgroups, Microvirga and Enterovirga, are shown in grey.

A recent study (Leducq et al. 2022) that analyzed 384 core genes extracted from 189 PPFM genomes identified four monophyletic lineages (A, B, C, and D) characterized by variations in genome size, GC content, gene content, and genome architecture. This study also found that horizontal gene transfer and incomplete lineage sorting significantly influence phylogeny inference. Moreover, they identified 45 new candidate species in addition to previously described species. Therefore, the genera encompass a far greater diversity of species than previously known. PPFM species are quite versatile and can be found in various environments. In Figure 1, we show their phylogeny and the first source of isolation of PPFM type strains. Leducq et al. (2022) reported that PPFMs from groups B and C were isolated from plants, sediments, soil, or water samples, often in association with anthropogenic environments, while species from groups A and D were mostly isolated from plants, especially the phyllosphere. Group C has been suggested to be close to the ancestor of the PPFMs, characterized with soil-related isolation sources, larger genome size, higher GC contents, and lack of calcium-dependent *mxaF*-type methanol dehydrogenase gene in many type strains (Alessa et al. 2021; Alleman et al. 2025; Leducq et al. 2022).

## Species-level interaction specificity

Mizuno et al. (2012) reported that PPFM colonization density was different among leaves of various vegetables grown in the same field, and particularly green perilla leaves even from different locations carried abundant PPFMs. The isolates from green and red perilla were limited to *Mb. fujisawaense* and *Mb. radiotolerans*. Tani et al. (2012) applied the whole-cell MALDI-TOF/MS analysis to assess the phylogenetic diversity of PPFM isolates collected from various plants and found that the strains belonging to *Mr. extorquens*, *Mb. adhaesivum*, *Mb. marchantiae*, *Mb. komagatae*, *Mb. brachiatum*, *Mb. radiotolerans*, and novel lineages close to *Mb. adhaesivum*, many of which were isolated from bryophytes, were found to be the most frequent phyllospheric colonizers. In a study conducted in the same field but across different seasons (rice in summer and barley in winter), PPFM isolates from various barley cultivars belonged only to *Mb. platani* and *Mb. goesingense*, whereas those from rice belonged to various species, including *Mb. aquaticum*, *Mb. tarhaniae*, *Mb. platani*, *Mb. fujisawaense*, *Mb. oryzae*, *Mb. radiotolerans*, *Mb. aerolatum*, *Mb. komagatae*, and *Mr. suomiense*. Even when collected from the same experimental field, PPFM isolates varied depending on the plant species and their respective cultivation seasons (Tani et al. 2015). On the other hand, most PPFMs isolated from plants are auxotrophic for pantothenate or β-alanine as its precursor, and these compounds were found in the *A. thaliana* leaf suspensions (Yoshida et al. 2019). The vitamin auxotrophy is not restricted to PPFMs; up to half of the strains of the bacteria isolated from *A. thaliana* leaves showed auxotrophy for biotin, niacin, pantothenate, and/or thiamine (Ryback et al. 2022). Thus, there is a species-level specificity in the interaction between plant and PPFM species, which can be dependent on the selection by plant and environmental factors or the adaptability of PPFMs.

## Methanol released by plants and its uptake by PPFMs

The prevalence of PPFMs in plants is closely tied to the availability of methanol, a carbon compound they efficiently utilize as a carbon and energy source. Methanol is naturally released during plant growth processes, primarily as a byproduct of cell wall remodeling and pectin demethylation associated with structural changes in roots and leaves during their expansion (Fall and Benson 1996; Knief et al. 2012). During growth, as new cell wall material is integrated into the expanding apoplast, the chemical bonds between macromolecules like pectin and cellulose must be cleaved and re-crosslinked. Pectin methylesterases (PMEs) are ubiquitously expressed in the cell wall and catalyze the removal of methyl groups from galactosyluronate methyl esters of pectins, releasing protons and methanol into the media (Frenkel et al. 1998).

Methanol production is influenced by the stage of leaf development and by temperature and plant hydraulics. The determination of free methanol in bean leaf extracts evidenced that the emission rate was considerably greater in young leaves than in mature ones (Nemecek-Marshall et al. 1995). This is in accordance with Corpe (1985), who reported that dead and abscised leaves exhibit a marked reduction in methanol production together with a marked reduction in PPFMs in the leaf impression method. Most of the methanol produced within the bean leaves was primarily emitted through the stomata, and the methanol emission rate decreased when the stomata were induced to close (Nemecek-Marshall et al. 1995). Later, Kawaguchi et al. (2011) reported that methanol was not limited to the area around the stomata and that high concentrations of free methanol were present in all areas of the leaf surface. These authors also observed that the concentration of free methanol on the living *Arabidopsis* leaf surfaces was equivalent to 25 mM methanol added in solid agar media but was ten times higher in wilting and dying plants. ^13^C-labeling studies with detached poplar (*Populus trichocarpa*) branches demonstrated a strong connection between photosynthetic carbon assimilation and the release of methanol during growth and development. *P. trichocarpa* leaf and branch gas exchange patterns in a field experiment indicated that temperature and hydraulic controls affect diurnal growth and methanol emissions. Methanol release was highly responsive to light and strongly dependent on temperature. During the accelerated growth phase that occurs between midnight and midday, growth and methanol increased positively with temperature, and during the decelerated growth phase between midday and midnight, growth and methanol emissions decreased with temperature constrained by leaf water stress (Jardine et al. 2024).

Using the methanol-reporter yeast *Candida boidinii* on *A. thaliana* plants, it was shown that the expression of methanol-inducible genes was highest in the dark period and lowest in the light period (Kawaguchi et al. 2011). Thus, it was postulated that the methanol concentration at the phyllosphere changes dynamically during the daily light-dark cycle. The volatiles once emitted will be diffused and unavailable for epiphytic microorganisms, thus, methylotrophs utilize methanol leaked through the plant cell wall in the dark. ^14^C-methanol tracing studies have revealed substantial methanol consumption in both the rhizosphere and phyllosphere of living plant material (Kanukollu et al. 2022). Abanda-Nkpwatt et al. (2006) demonstrated the consumption of methanol by *Mr. extorquens* strain ME4; they encountered significantly lower levels of methanol emission when *Nicotiana tabacum* seedlings were co-cultivated with this strain.

*Mr. extorquens* strain AM1 is a key model organism for studying methylotrophic metabolism in *Alphaproteobacteria*. Through multi-omics studies, its methylotrophic metabolism has been extensively studied physiologically and biochemically (Ochsner et al. 2015). The oxidation of methanol to formaldehyde is catalyzed by two pyrroloquinoline quinone (PQQ)-dependent methanol dehydrogenases (MDHs), MxaFI and XoxF, that utilize calcium (Ca^2+^) or lanthanide (Ln^3+^) as cofactors (Keltjens et al. 2014; Skovran et al. 2019). MxaF and XoxF share about 50% amino acid sequence identity, and only MxaF is associated with its beta-subunit, MxaI. XoxF is the first Ln-dependent enzyme to be known (Hibi et al. 2011; Pol et al. 2014), and the MxaF/XoxF expression is switched depending on the availability of Ln (Ln switch, Vu et al. 2016). The formaldehyde is then oxidized to formate in the bacterial cytoplasm for energy generation in the pathway dependent on the cofactor tetrahydromethanopterin and further to CO_2_ by several formate dehydrogenases. Formate can also be conjugated with tetrahydrofolate to form methylenetetrahydrofolate, which is a substrate of the serine cycle for carbon assimilation (Sy et al. 2005). The serine cycle is associated with the tricarboxylic acid cycle and the ethylmalonyl-CoA pathway that regenerates glyoxylate to supply C_2_ units for the serine cycle. Methylamine can be oxidized by methylamine dehydrogenase to form formaldehyde (Chistoserdova et al. 2003). The methylotrophic pathway revealed within *Mr. extorquens* AM1 can be found in most PPFMs. However, methylamine utilization is not common in PPFMs (all group B strains can utilize it). Some species lack the *mxa* gene cluster, therefore, they cannot grow on methanol in the absence of Ln but can grow in the presence of Ln. Formate dehydrogenases among the known 4 types can also be lacking in some species (Alessa et al. 2021). Thus, methylotrophy can be a hallmark of the PPFMs, but such diversity can be seen by comparative genomics.

PPFMs can co-metabolize methanol alongside other alternative carbon and nitrogen sources that are leached from the plant surfaces (Kwak et al. 2014; Schauer and Kutschera 2011; Sy et al. 2005). However, methylotrophy is crucial for symbiosis. Both types of MDHs are highly expressed in PPFMs in the phyllosphere of naturally growing plants (Delmotte et al 2009). MDHs make the bacteria advantageous compared to the respective mutants (Schmidt et al. 2010; Sy et al. 2005). *Mr. extorquens* growing on plant surfaces upregulate genes involved in methylotrophic metabolism, such as *mxaF* and *fae* (formaldehyde activating enzyme) (Gourion et al. 2006). In the interaction between *Crotalaria podocarpa* and *Mb. nodulans* strain ORS 2060, it was demonstrated that the *mxaF* of *Mb. nodulans* is expressed in the root nodules, particularly at the nodule apex. This localization may be driven by the presence of methanol produced during nodule development. Furthermore, the loss of methylotrophic activity disrupted the symbiosis with *C. podocarpa* (Jourand et al. 2005). In *Mb. aquaticum* strain 22A, three chemoreceptors (*mtpA*, *mtpB*, and *mtpC*) were identified as key mediators of methanol taxis. Interestingly, the taxis operated by *mtpB* and *mtpC* are dependent on XoxF and MxaF activities, respectively, whereas the ligand for MtpA was suggested to be formaldehyde. A mutant strain lacking all three receptors showed impaired methanol taxis and reduced ability to colonize plant surfaces, suggesting that methylotrophy is also crucial to locating the plants (Tani et al. 2023).

Pathogenic bacteria and fungi secrete cell wall-degrading enzymes, such as pectin methylesterases (PMEs), to facilitate successful plant infection (Wormit and Usadel 2018), releasing methanol as a byproduct. A BLAST search using the PME from *Erwinia chrysanthemi* as a query against the available PPFM genomes at NCBI yielded no hits. However, a gene encoding a putative pectin acetylesterase (PAE), which hydrolyzes the acetyl esters of pectin, was identified in *Mb. oryzae* strain CBMB20 (Kwak et al. 2014). Since the deacetylation of pectin can improve the accessibility of PMEs and other cell-wall-degrading enzymes (Bonnin et al. 2003), Kwak et al. (2014) suggested that strain CBMB20 can actively acquire nutrients from the plants. Moreover, Jourand et al. (2005) observed significant tissue disorganization in the apical region of nodules colonized by *Mb. nodulans* and hypothesized that this disruption could result from the production of pectinases by either the plant or the bacteria. A BLASTp search with *Mb. oryzae* PAE (AIQ87844.1) against the PPFM group retrieved hits (99.7–59% identity and 100–87% query coverage) in various PPFM genomes. It is compelling to suggest that PAEs could function as a mechanism by which PPFMs facilitate the extraction of methanol from plant cell walls.

## Plant-associated lifestyle

The rhizosphere and phyllosphere are dynamic microenvironments that surround a plant’s roots and aboveground tissues, respectively. Both regions support a rich diversity of microorganisms, being influenced by plant secretions (reviewed in Antoszewski et al. 2022). The composition of bacterial communities in these environments is shaped by the unique characteristics of each microhabitat. The phyllosphere is an unstable environment, with harsh conditions such as temperature shifts, UV radiation, drought stress, and nutrient limitations (Lindow and Brandl 2003; Vorholt 2012). In contrast, the rhizosphere is more buffered, with fewer fluctuations in environmental factors. Microbial communities in both zones can establish mutualistic relationships with plants in two primary ways: as endophytes, which colonize internal plant tissues, or as epiphytes, which reside on external surfaces. The endophytic microbiome is typically less diverse than those in the rhizosphere or phyllosphere due to the selective nature of the plant’s internal environment, which is influenced by immune defenses and limited nutrient availability (Bulgarelli et al. 2013; Müller et al. 2016).

PPFMs utilize diverse adaptation mechanisms to thrive on plant surfaces. *Mr. extorquens* AM1 was found to require the 2-component response regulator PhyR (phyllosphere-induced regulator) for successful plant surface colonization (Gourion et al. 2006). PhyR targets 246 regulons, including genes associated with resistance to heat shock, desiccation, oxidative stress, UV radiation, ethanol, and osmotic stress (Gourion et al. 2008). A mutant of *Mr. extorquens* DM4 lacking hopanoids—sterol-like membrane lipids that help maintain membrane stability and rigidity (Belin et al. 2018)—showed small colonies and increased sensitivity to osmotic and pH stress, as well as a range of toxins (Bradley et al. 2017). Delmotte et al. (2009), using a proteogenomics approach, observed that proteins related to stress (eg, superoxide dismutase, chaperonins, cold-shock proteins) were abundantly expressed in PPFMs associated with the phyllosphere of soybean, clover, and Arabidopsis. Alamgir et al. (2015) found that many *Methylobacterium* species produce the antioxidative amino acid ergothioneine, which helps them survive under heat, UV, and sunlight stress. In addition to producing pink carotenoid pigments, *Methylobacterium* also contains specific UVA-absorbing compounds, such as avobenzone, that enable them to withstand harmful UV-A radiation (Yoshida et al. 2017). Moreover, *Mr. extorquens* strain AM1 contains two KaiC proteins that contribute to resistance against temperature and UV light. These proteins help regulate cellular responses, enabling strain AM1 cells to adapt to fluctuating environmental conditions (Iguchi et al. 2018). Biofilms can also help bacteria resist challenging environmental fluctuations; two endophytic strains, *Mb. mesophilicum* SR1.6/6 and *Mr. extorquens* AR1.6/2 were able to form biofilm covering the sugarcane roots (Rossetto et al. 2011). Furthermore, *Mb. aquaticum* strain 22A produces a staphyloferrin B-like siderophore that is essential for Ln solubilization, biofilm formation, and enhancing the strain’s stress tolerance (Juma et al. 2022).

Samples of the moss *Funaria hygrometrica* collected from the field revealed that PPFMs colonized the surface of adult plants, preferentially inhabiting the grooves between adjacent epidermal cells, especially in areas where the cuticle was thin (Hornschuh et al. 2002). Two GFP-tagged *Methylobacterium* isolates (Mb49 and HSC5) were observed colonizing clover leaf surfaces, where they formed large aggregates and infiltrated deep into epidermal grooves, though they did not penetrate the lower tissue layers (Omer et al. 2004). GFP-tagged *Mr. extorquens* strain Rab1 (DSM 21961) inoculated in strawberry plants demonstrated extensive colonization of both the upper and lower leaf surfaces, with a significant accumulation of bacterial cells on the trichomes (Verginer et al. 2010). When GFP-tagged *Mr. suomiense* CBMB120 was inoculated into rice and tomato seeds, bacterial cells were visibly present in the substomatal chambers of the leaves. However, intracellular colonization was observed only in the surface-sterilized root sections of tomato (Poonguzhali et al. 2008). Though *Methylobacterium* sp. 2A-mVenus was mainly observed on the surface of *A. thaliana* primary and lateral roots; a small subset was observed to colonize the apoplast of primary root cells, suggesting its facultative endophytic nature (Grossi et al. 2024). *Mr. extorquens* was detected in the needle mesophyll and parenchymatous cells of the stems and in the root cortex and xylem of inoculated pine seedlings (Pohjanen et al. 2014). In the microalgae *Scenedesmus vacuolatus* and *Haematococcus lacustris* inoculated with *Mb. goesingense* strain Vab1, abundant bacterial biofilm-like assemblages were observed on the algal surfaces within the phycosphere (Krug et al. 2020). Furthermore, *Methylobacterium* species were also found as an intracellular symbiont. Koskimäki et al. (2015) demonstrated that *Mr. extorquens* DSM 13060, whose genome encodes numerous eukaryote-like genes, aggregates near the nucleus of Scots pine shoot cells, suggesting that the bacterium targets the symbiont’s nuclear processes, including gene expression and regulation.

## Plant growth-promoting (PGP) traits

**Figure 2:**
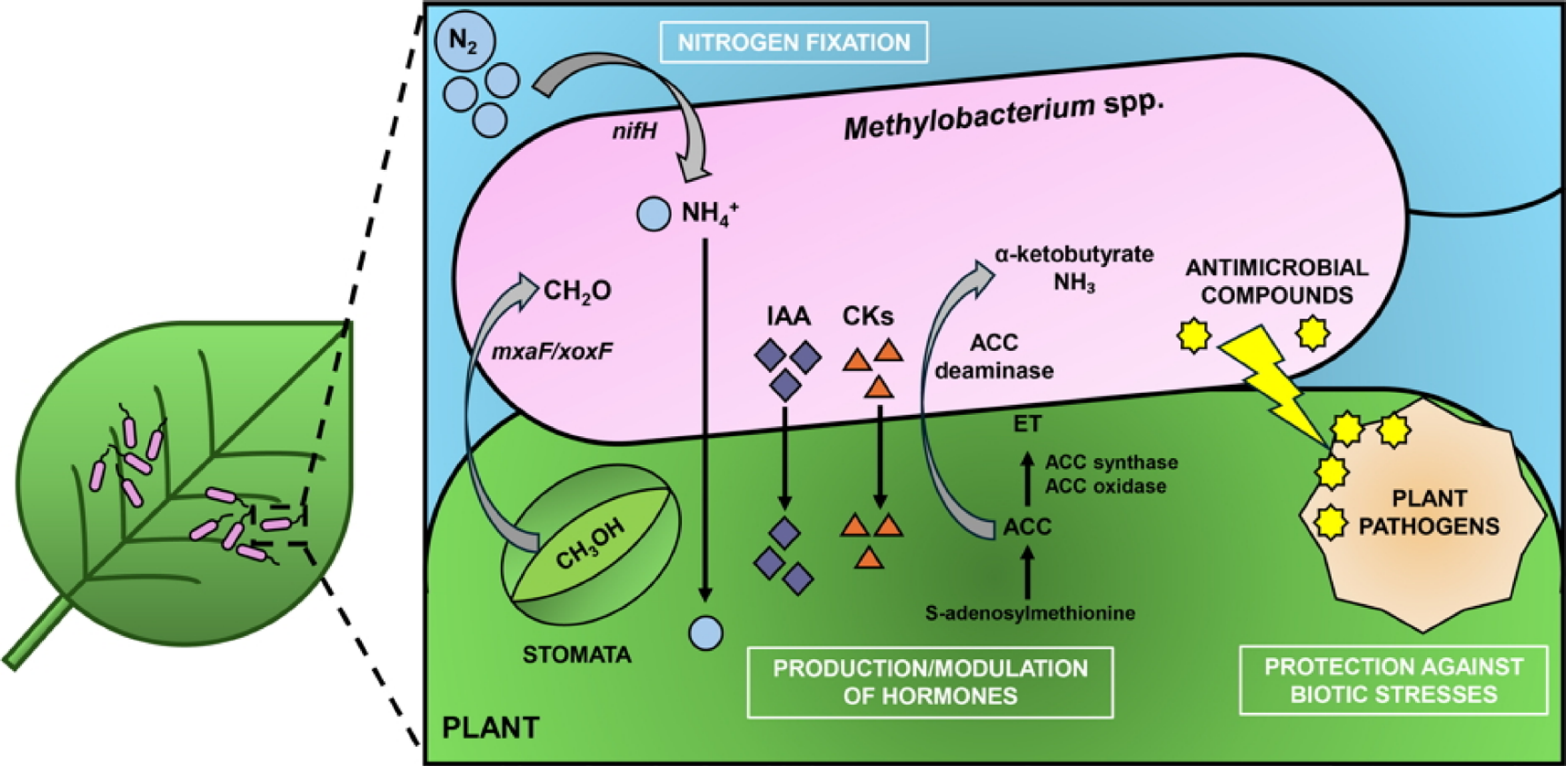
Figure 2. Interaction between *Methylobacterium* species and plants. PGP traits of *Methylobacterium* species in the phyllosphere include the production of IAA and CKs, modulation of ET, biological nitrogen fixation, and protection against plant pathogens. Methanol (CH3OH) released by plants through stomata is oxidized by *Methylobacterium* into formaldehyde (CH2O) through the action of the MDHs MxaFI and XoxF. Similar PGP traits occur in the rhizosphere.

Rather than being passive inhabitants, PPFMs actively interact with their plant hosts, promoting seed germination and enhancing plant growth through a range of biochemical and genetic mechanisms. This review highlights particular processes involved in plant growth promotion, including the production of phytohormones such as cytokinins (CKs) and indole-3-acetic acid (IAA), the modulation of ethylene (ET) via the degradation of its precursor ACC (1-aminocyclopropane-1-carboxylate), biological nitrogen fixation, and protection against plant pathogens (Madhaiyan et al. 2007; Ryu et al. 2006) (Figure 2).

### Production and/or modulation of growth-regulating hormones

PPFMs are capable of producing phytohormones. Particularly, CK plays a crucial role in a wide range of biological processes in plants, including cell division and elongation, apical dominance, shoot development, and nutrient uptake (Prasad 2022). It has been suggested that various PPFMs can produce CKs as by-products of tRNA degradation that are chemically identical to those naturally produced by plants. Through LC-MS analysis, Palberg et al. (2022) demonstrated that most PPFMs are capable of synthesizing cytokinins, with trans-zeatin (tZ) and 2-methylthio-zeatin being the dominant forms. Interestingly, CK production was higher when methanol, used as a carbon source in the growth medium, was present at lower concentrations (Palberg et al. 2022). Through the production of CK, they can stimulate plant cell division, which in turn leads to an increased release of methanol that PPFMs can utilize. One of the most active cytokinin forms produced by PPFMs is tZ, with the tRNA delta(2)-isopentenylpyrophosphate transferase (*miaA*) gene being responsible for its synthesis in *Mr. extorquens* (Koenig et al. 2002). The *miaA* is conserved in almost all PPFM genomes (Alessa et al. 2021).

IAA, the primary auxin in plants, plays a crucial role in regulating various physiological processes. The response of roots to exogenous IAA includes tropism and changes in the root architecture. Plant roots are sensitive to fluctuations in IAA concentrations; low concentrations of auxin stimulate root elongation, while higher concentrations inhibit it. As a result, the impact of microbially produced IAA on plants can vary, with effects ranging from beneficial to deleterious depending on the concentration (Ludwig-Müller 2015).

IAA production is strain-specific; different strains of the same species showed different levels of IAA production. Palberg et al. (2022) comment that the differential phytohormone production of some *Methylobacterium* strains may indicate specialization and adaptation for their respective ecological niches. Microbial biosynthesis of IAA can be categorized into tryptophan-dependent and tryptophan-independent pathways, depending on whether L-tryptophan (Trp) is used as a precursor. There are five main tryptophan-dependent pathways: the indole-3-acetamide (IAM) pathway, the indole-3-pyruvic acid (IPA/IPyA) pathway, the indole-3-acetonitrile (IAN) pathway, the tryptamine (TAM) pathway, and the tryptophan side-chain oxidase (TSO) pathway (extensively reviewed in Tang et al. 2023). *Mb. mesophilicum*, isolated from the phyllosphere of *Lolium perenne* L., has been shown to synthesize IAA from Trp via the IPA/IPyA pathway (Ivanova et al. 2001). Additionally, the genes responsible for the IAM and IAN pathways have been found in the genome of *Methylobacterium* sp. 2A (Grossi et al. 2020). Most members of clade C and some members of clade A of the genus *Methylobacterium* harbor the Trp decarboxylase, monoamine oxidase, and aldehyde dehydrogenase genes involved in one of the IAA biosynthesis pathways (Alessa et al. 2021).

The synthesis of Trp in microbial cells is energetically costly, so its endogenous concentration in microorganisms is often low. High levels of IAA are produced only when Trp is supplied exogenously in the culture medium (Patten et al. 2013). Specifically, strains of *Mb. fujisawaense* and *Mr. extorquens* produced significant amounts of IAA in vitro only when supplemented with Trp. Furthermore, their inoculation in canola plants resulted in elevated concentrations of both IAA and cytokinins in plant extracts (Madhaiyan et al. 2006). Similarly, treatment with *Methylobacterium* sp. strains CMBM20 and CMBM110 resulted in elevated levels of IAA and cytokinins in red pepper extracts (Ryu et al. 2006). In line with this, *Methylobacterium* sp. strain 2A produced high levels of IAA in vitro and impacted the root growth and architecture of *Arabidopsis thaliana* plants. Moreover, its inoculation enhanced the activity of the auxin-responsive *DR5* promoter and partially restored the gravitropic response in *A. thaliana yucQ* mutant plants (Grossi et al. 2024).

ET is a multifunctional gaseous phytohormone that regulates a wide range of plant developmental processes, including seed germination, flowering, fruit ripening, and senescence. It also plays a crucial role in plant responses to various environmental stresses, both biotic and abiotic (Iqbal et al. 2017; Khan et al. 2024). Stress factors that stimulate ethylene production include submergence, heat, shade, exposure to heavy metals, high salinity, and nutrient and water deficiencies. While ET is essential for plant adaptation to stress, elevated ET levels can negatively affect growth, leading to inhibited root elongation, premature abscission, and accelerated senescence. Auxin and ET act synergistically in the inhibition of primary root elongation and root hair formation (Muday et al. 2012; Qin et al. 2019; Růzicka et al. 2007) by modulating cell proliferation in the root apical meristem and cell elongation in the elongation zone (Růzicka et al. 2007; Street et al. 2015). Ethylene regulates root growth by affecting auxin biosynthesis, transport, and signaling. However, they can also have antagonistic effects in some areas of root development, such as lateral root formation. The interaction of ET and auxin is reciprocal, as auxin can induce ET biosynthesis and signaling (Muday et al. 2012).

In ET biosynthesis, the precursor of the ET hormone is aminocyclopropane-1-carboxylic acid (ACC), which is derived from S-adenosylmethionine. ACC is then converted to ethylene by ACC synthase (ACS) and ACC oxidase (Dubois et al. 2018). These enzymes are transcriptionally regulated by biotic and abiotic factors (Khan et al. 2024). Modification of ET biosynthesis or signaling resulted in increased yield in crops. *Zea mays ACS6* RNAi lines with reduced ethylene biosynthesis and sensitivity showed a significant increase in grain yield when exposed to drought stress in the field (Habben et al. 2014). Similarly, under root-lodging conditions, maize plants with increased expression levels of *ARGOS8* (an auxin-regulated gene involved in organ size that negatively regulates the ethylene response) exhibited increased yield (Shi et al. 2017). Several rhizobacteria can utilize ACC as a nitrogen source through the action of the enzyme ACC deaminase that catalyzes the conversion of ACC into ammonia (NH_3_) and α-ketobutyrate, enabling the bacteria to assimilate nitrogen while simultaneously reducing ethylene levels in plants. Plants that grow in association with ACC deaminase-containing rhizobacteria generally have longer roots and shoots and are more resistant to growth inhibition by a variety of ethylene-inducing stresses (Glick 2014). Moreover, it was identified in the genome of *Mb. oryzae* strain CBMB20 (*accD*; MOC_1898; Kwak et al. 2014). Inoculation of canola seeds with this strain led to a reduction in ACC concentration in root exudates and increased the root length of seedlings (Madhaiyan et al. 2007). In contrast, inoculation with *Mr. extorquens* strain CBMB120, which lacks ACC deaminase activity (Madhaiyan et al. 2006), resulted in ACC exudation levels similar to those of the control (Madhaiyan et al. 2007). Moreover, canola seeds treated with *Mb. fujisawaense* strains exhibiting ACC deaminase activity accumulated low levels of ACC in their tissues and prevented the inhibition of root elongation (Madhaiyan et al. 2006). Under greenhouse conditions, red pepper plants inoculated with *Mb. oryzae* strain CBMB20 and *Mb. fujisawaense* strain KACC10744 which possesses both ACC deaminase and IAA activity, led to improved average nodal length and specific leaf weight (Hari et al. 2010). *Mb. oryzae* strain CBMB20 and *Mr. suomiense* strain CBMB120 were inoculated in co-aggregation with *Azospirillum brasilense* strain CW903 in tomato plants under water stress conditions. Both combinations significantly reduced ethylene levels, helping mitigate the effects of water stress (Joe et al. 2014). In another study, when two rice cultivars were exposed to salt stress and treated with *Mb. oryzae* strain CBMB20, there was a reduction in ACC accumulation and ACC oxidase activity, along with an improvement in vacuolar H^+^-ATPase activity in the plants (Chatterjee et al. 2019). The gene encoding ACC deaminase (*acdS*) was identified in the genome of *Methylobacterium* sp. 2A, enabling the bacterium to utilize ACC as its sole nitrogen source for growth (Grossi et al. 2024). In addition, *Mb. symbioticum*, isolated from spores of the symbiotic fungus *Glomus iranicum* (Pascual et al. 2020), exhibited ACC-deaminase activity, which contributed to prolonged canopy stay-green and enhanced photosynthetic efficiency in wheat (Valente et al. 2024).

### Biological nitrogen fixation

Nitrogen is a key limiting factor for plant growth in most agroecosystems, as atmospheric nitrogen (N_2_) is not directly available for plant metabolism. However, nitrogen-fixing bacteria can convert N_2_ into ammonium ion (NH_4_^+^) through the action of the nitrogenase enzyme, which is encoded by the *nif* genes. Plants can readily assimilate ammonium ion to produce nitrogenous biomolecules. Nitrogen-fixing bacteria can live freely or symbiotically in specialized structures known as nodules (Sun et al. 2021).

*Mb. nodulans* strain ORS2060 was the first *Methylobacterium* species found to form nodules. It contains both a *nifH* gene and a *nodA* gene, the latter encoding an acyltransferase involved in Nod factor biosynthesis. The *nodA* gene was closely related to that of *Bradyrhizobium*, indicating it was likely acquired via horizontal gene transfer (Sy et al. 2001). Jaftha et al. (2002) characterized nine isolates that nodulate roots of *Lotononis bainesii* that were closely related to *Mb. nodulans* (98% similarity 16S rRNA gene). The facultative methylotrophic nature of these isolates was also demonstrated by their ability to grow in the presence of methanol as a sole carbon source. Jourand et al. (2004) proposed that 72 non-pigmented bacterial strains that specifically induce nitrogen-fixing root nodules on the legume species *Crotalaria glaucoides*, *C. perrottetii*, and *C. podocarpa* form a homogeneous group that is separate from other legume root-nodule-forming bacteria and belong to the genus *Methylobacterium*. They can grow on C1 compounds and carry *mxaF* and *nodA* genes. Similarly, *Methylobacterium* sp. strain NPFM-SB3 isolated from *Sesbania rostrata* stem nodules possessed nitrogenase activity and *nodA* genes (Senthilkumar et al. 2009). This strain induced nodule-like structure in rice, and the entry of bacteria occurred at the points of lateral root emergence. Raja et al. (2006) screened 250 *Methylobacterium* isolates from vegetable crops for nitrogen fixation and found 11 that could grow on nitrogen-free medium, with 10 testing positive for the *nodA* gene. In addition, *Methylobacterium* sp. strain MV10, which possesses a functional *nifH* gene but lacks the *nodA* gene, was unable to form nodules in *Crotalaria* sp. Madhaiyan et al. (2009) isolated two *Methylobacterium* strains, CMCJ317 and CMSA322, that contain the *mxaF* and *nodA* genes. Both strains exhibited high nitrogenase activity and were capable of forming nodules on *Crotalaria juncea* and *Macroptilium atropurpureum*, thereby enhancing nitrogen concentration in the shoots.

Aside from these nodule-forming *Methylobacterium*, other members of the group fix nitrogen. Madhaiyan et al. (2015) identified an efficient nitrogen-fixing strain, L2-4, close to *Mb. radiotolerans*, from surface-sterilized leaf tissues of *Jatropha curcas*. This strain possesses the *nifH* gene and exhibits nitrogenase activity in vitro. When reinoculated into *J. curcas*, it resulted in improved plant height, leaf number, chlorophyll content, and stem volume (Madhaiyan et al. 2015). *Mb. symbioticum* SB0023/3^T^ reduced the need for chemical nitrogen fertilizers in maize, rice, and wine grapes, resulting in higher yields. The SPAD (Soil and Plant Analysis Development) value, which correlates with chlorophyll content in leaves, was significantly higher in plants inoculated with this strain than in non-inoculated ones. This result demonstrates its ability to maintain the chlorophyll content and the nutritional status while reducing nitrogen fertilization (Pascual et al. 2020). In a similar way, *Mb. symbioticum* also reduced nitrogen application in maize and strawberry crops while enhancing the photosynthetic capacity (Torres Vera et al. 2024). In a recent study, Valente et al. (2024) applied *Mb. symbioticum* foliarly to wheat and found that inoculation increased root length density, delayed leaf senescence, extended photosynthetic activity, and enhanced stomatal conductance and PSII efficiency under reduced nitrogen doses. However, Arrobas et al. (2024) and Rodrigues et al. (2024) evaluated the impact of the commercial product BlueN® (Corteva Agriscience, Indianapolis, IN) which contains *Mb. symbioticum* cells, on lettuce and maize growth, respectively, and found that the plants showed a stronger response to nitrogen fertilization than to the inoculation of the bacteria. They concluded that the beneficial effects of *Mb. symbioticum* does not extend to all crops, as a high level of specificity between the microorganism and the host plant is required.

Notably, within the type strains of PPFMs, nitrogenase genes responsible for nitrogen fixation have been identified only in *Mb. nodulans*. The genome sequences of the following strains were found to contain nitrogenase genes as revealed by our search in the NCBI genome database: *Methylobacterium* sp. WSM2598 (Ardley et al. 2014) and *Methylobacterium* sp. 4-46 (Ardley et al. 2009) isolated from *Lotononis bainesii*, and *Methylobacterium* sp. CB376 (a synonym of strain 4-46; Fleischman and Kramer 1998). The genome sequences of the other above-mentioned strains, which tested positive for *nodA* and *nifH* and for assay of nitrogenase and nodulation, are not publicly available.

### Protection against biotic stresses

PGPR can confer resistance or tolerance to the host plant from biotic stresses through direct mechanisms, such as the release of antimicrobial compounds (e.g., siderophores, antibiotics, hydrolytic enzymes, and other secondary metabolites), and indirect mechanisms which are related to the competition with pathogens for space and nutrients and their ability to modulate plant defense responses or by inducing systemic resistance (ISR) (Kumar et al. 2021).

Alessa et al. (2021) examined the antifungal activity of PPFMs; species from clade A exhibited strong antifungal activity, those from clade B showed moderate activity, and most strains in clade C demonstrated a clear suppressive effect on *Fusarium* growth. *Methylobacterium* sp. 2A demonstrated biocontrol activity against *Phytophthora infestans, Botrytis cinerea*, and *Fusarium graminearum* in dual confrontation assays. Genomic analysis suggested that these PPFMs could inhibit the growth of pathogenic microbes through the action of antimicrobial compounds such as chitinase, cellulase, pectinase, secondary metabolite, and siderophores (Alessa et al. 2021; Grossi et al. 2020; Kwak et al. 2014). Further investigation is required to identify which secondary metabolites of PPFMs target each particular pathogen.

Different studies have demonstrated that PPFM inoculation induces defense enzymes, such as phenylalanine ammonia-lyase (PAL), peroxidase, β-1,3-glucanase, and chitinase, enhances plant protection, and reduces disease symptoms. For example, rice and cotton plants inoculated with *Methylobacterium* sp. strain PPFM-Os-07 or *Mr. extorquens* strain CO47, respectively, and infected with *Rhizoctonia solani*, exhibited reduced disease symptoms and increased defense responses (Madhaiyan et al. 2004; Poorniammal et al. 2009). Similarly, tomato plants inoculated with *Methylobacterium* species showed decreased symptoms of *Ralstonia solanacearum* infection and elevated levels of PR proteins associated with ISR (Yim et al. 2013).

*Methylobacterium* sp. 2A showed efficacy in reducing disease symptoms in greenhouse potato plants infected with *Phytophthora infestans* (Grossi et al. 2020). Additionally, *Mr. rhodesianum* strain M520 significantly reduced *Meloidogyne incognita* nematode invasion and suppressed infection in cucumber roots (Zhao et al. 2023). Several PPFMs (*Mb. indicum*, *Mb. komagatae*, *Mb. aerolatum*, and *Mr. rhodinum*) were enriched in healthy rice leaf samples and significantly mitigated symptoms of *Xanthomonas oryzae* pv. *oryzae* infection (Oeum et al. 2024). Furthermore, *Methylobacterium* sp. P1-11 promoted the growth of the beneficial fungal endophyte *Serendipita indica* and facilitated its plant colonization. Tomato plants co-inoculated with *S. indica* and strain P1-11 exhibited significantly reduced symptoms caused by *Fusarium oxysporum* (del Barrio-Duque et al. 2020).

## Significance of PPFMs in sustainable agriculture

The reported positive effects of PPFMs on plant physiology prompted us to conduct a search against the genomes of all PPFM type strains described to date. We used as queries the genes of *Mr. extorquens* AM1/PA1, *Mb. oryzae* CBMB20^T^, *Mb. nodulans* ORS 2060^T^, and *Mb. aquaticum* 22A, which encode proteins involved in methylotrophy and PGP traits (Supplementary Table S1). The table suggests that PPFM-based microbial inoculants could be promising tools for improving crop performance, offering a cost-effective and technically efficient alternative. Examples of PPFM-based inoculants that were effective for maize and strawberry crops include *Mb. symbioticum* and *Mr. extorquens* strain Rab1. Several patents have been granted for the application of PPFMs in enhancing plant growth, yield, seed germination, male fertility, fruit production, nutritional qualities, and in controlling root lesion nematodes (e.g., US6174837B1; US6329320B1; US7435878B2; US12108765B2; US11871753B2). Additionally, these bacteria have been demonstrated to enhance the yield of cultivated algae (e.g., US20110269219A1), suggesting their potential applicability in algae-derived biofuel production.

## Future perspectives

The ability of PPFMs to enhance plant tolerance to various environmental stresses makes them particularly relevant in the context of climate change. Their biocontrol potential is another avenue of interest; some species produce antifungal metabolites and ISR in plants, offering protection against pathogens. This dual role as a biofertilizer and biocontrol agent positions them as a sustainable alternative to chemical inputs in agriculture. Beyond its agricultural applications, PPFMs play a significant role in the global carbon cycle. By oxidizing methanol, one of the most abundant volatile organic compounds in the atmosphere, these bacteria contribute to carbon regulation and energy flow in ecosystems. Their presence in extreme environments, such as Antarctic soils and arid regions, highlights their adaptability and ecological significance.

The use of PPFM-based bioinoculants aligns with the goals of sustainable agriculture by reducing dependency on chemical fertilizers and pesticides. These bioinoculants not only enhance crop yields but also promote soil health and environmental sustainability. By combining traditional microbiology with modern omics technologies, researchers can unlock the full potential of this genus, contributing to food security and environmental conservation. Moving forward, research should focus on several key areas:

• Understanding the molecular mechanisms underlying the PPFM-plant interaction, particularly the signaling pathways involved in plant growth promotion.
• Investigating the potential synergistic effects between PPFMs and other beneficial microorganisms in the plant microbiome.
• Developing practical applications through the optimization of strain selection and delivery methods for agricultural use.
• Exploring their potential role in improving crop yield and quality under adverse conditions, while maintaining focus on the safety and stability of PPFM-based agricultural products. The study of the diversity of PPFMs in the environment would provide additional insights. We propose this as an important direction for future research to better understand their ecological adaptations.

## Abbreviations

ACC: aminocyclopropane-1-carboxylic acid
ACS: ACC synthase
CK: cytokinin
ET: ethylene
GFP: green fluorescent protein
IAA: indole-3-acetic acid
IAM: the indole-3-acetamide pathway
IAN: the indole-3-acetonitrile pathway
IPA/IPyA: the indole-3-pyruvic acid pathway
ISR: induced systemic resistance
MDH: methanol dehydrogenase
*miaA*: tRNA delta(2)-isopentenylpyrophosphate transferase gene
PAE: pectin acetylesterase
PAL: phenylalanine ammonia-lyase
PGP: plant growth-promoting
PGPR: plant growth-promoting rhizobacteria
PME: pectin methylesterase
PPFM: pink-pigmented facultative methylotroph
Trp: L-tryptophan
tZ: trans-zeatin

## Description of supplementary files

Supplementary Table S1. The genes involved in methylotrophy and plant growth promotion in PPFM type strains.

## References

[RAbanda-Nkpwatt2006] Abanda-Nkpwatt D, Müsch M, Tschiersch J, Boettner M, Schwab W (2006) Molecular interaction between *Methylobacterium extorquens* and seedlings: Growth promotion, methanol consumption, and localization of the methanol emission site. *J Exp Bot* 57: 4025–4032 17043084 10.1093/jxb/erl173

[RAlamgir2015] Alamgir KM, Masuda S, Fujitani Y, Fukuda F, Tani A (2015) Production of ergothioneine by *Methylobacterium* species. *Front Microbiol* 6: 1185 26579093 10.3389/fmicb.2015.01185 PMC4621440

[RAlessa2021] Alessa O, Ogura Y, Fujitani Y, Takami H, Hayashi T, Sahin N, Tani A (2021) Comprehensive comparative genomics and phenotyping of *Methylobacterium* species. *Front Microbiol* 12: 740610 34737731 10.3389/fmicb.2021.740610 PMC8561711

[RAlleman2025] Alleman AB, Stolyar S, Marx CJ, Leducq JB (2025) Led astray by 16S rRNA: Phylogenomics reaffirms the monophyly of *Methylobacterium* and lack of support for *Methylorubrum* as a genus. *ISME J* 19: wraf011 39834026 10.1093/ismejo/wraf011 PMC11833323

[RAntoszewski2022] Antoszewski M, Mierek-Adamska A, Dąbrowska GB (2022) The importance of microorganisms for sustainable agriculture: A review. *Metabolites* 12: 1100 36422239 10.3390/metabo12111100 PMC9694901

[RArdley2014] Ardley J, Tian R, Howieson J, Yates R, Bräu L, Han J, Lobos E, Huntemann M, Chen A, Mavromatis K, et al. (2014) Genome sequence of the dark pink pigmented *Listia bainesii* microsymbiont *Methylobacterium* sp. WSM2598. *Stand Genomic Sci* 9: 5 25780498 10.1186/1944-3277-9-5 PMC4334988

[RArdley2009] Ardley JK, O’Hara GW, Reeve WG, Yates RJ, Dilworth MJ, Tiwari RP, Howieson JG (2009) Root nodule bacteria isolated from South African *Lotononis bainesii, L. listii* and *L. solitudinis* are species of *Methylobacterium* that are unable to utilize methanol. *Arch Microbiol* 191: 311–318 19152052 10.1007/s00203-009-0456-0

[RArrobas2024] Arrobas M, Correia CM, Rodrigues MÂ (2024) *Methylobacterium symbioticum* applied as a foliar inoculant was little effective in enhancing nitrogen fixation and lettuce dry matter yield. *Sustainability (Basel)* 16: 4512

[RBassalik1913] Bassalik K (1913) Über die Verarbeitung der Oxalsäure durch *Bacillus extorquens* n. sp. *Jahrb Fur Wiss Bot* 53: 255–302

[RBelin2018] Belin BJ, Busset N, Giraud E, Molinaro A, Silipo A, Newman DK (2018) Hopanoid lipids: From membranes to plant-bacteria interactions. *Nat Rev Microbiol* 16: 304–315 29456243 10.1038/nrmicro.2017.173 PMC6087623

[RBonnin2003] Bonnin E, Le Goff A, van Alebeek GWM, Voragen AGJ, Thibault JF (2003) Mode of action of *Fusarium moniliforme* endopolygalacturonase towards acetylated pectin. *Carbohydr Polym* 52: 381–388

[RBradley2017] Bradley AS, Swanson PK, Muller EE, Bringel F, Caroll SM, Pearson A, Vuilleumier S, Marx CJ (2017) Hopanoid-free *Methylobacterium extorquens* DM4 overproduces carotenoids and has widespread growth impairment. *PLoS One* 12: e0173323 28319163 10.1371/journal.pone.0173323 PMC5358736

[RBulgarelli2013] Bulgarelli D, Schlaeppi K, Spaepen S, van Themaat EVL, Schulze-Lefert P (2013) Structure and functions of the bacterial microbiota of plants. *Annu Rev Plant Biol* 64: 807–838 23373698 10.1146/annurev-arplant-050312-120106

[RChatterjee2019] Chatterjee P, Kanagendran A, Samaddar S, Pazouki L, Sa TM, Niinemets Ü (2019) *Methylobacterium oryzae* CBMB20 influences photosynthetic traits, volatile emission and ethylene metabolism in *Oryza sativa* genotypes grown in salt stress conditions. *Planta* 249: 1903–1919 30877435 10.1007/s00425-019-03139-w PMC6875431

[RChistoserdova2003] Chistoserdova L, Chen S-W, Lapidus A, Lidstrom ME (2003) Methylotrophy in *Methylobacterium extorquens* AM1 from a genomic point of view. *J Bacteriol* 185: 2980–2987 12730156 10.1128/JB.185.10.2980-2987.2003 PMC154073

[RCorpe1985] Corpe WA (1985) A method for detecting methylotrophic bacteria on solid surfaces. *J Microbiol Methods* 3: 215–221

[RdelBarrio-Duque2020] del Barrio-Duque A, Samad A, Nybroe O, Antonielli L, Sessitsch A, Compant S (2020) Interaction between endophytic Proteobacteria strains and *Serendipita indica* enhances biocontrol activity against fungal pathogens. *Plant Soil* 451: 277–305

[RDelmotte2009] Delmotte N, Knief C, Chaffron S, Innerebner G, Roschitzki B, Schlapbach R, von Mering C, Vorholt JA (2009) Community proteogenomics reveals insights into the physiology of phyllosphere bacteria. *Proc Natl Acad Sci USA* 106: 16428–16433 19805315 10.1073/pnas.0905240106 PMC2738620

[RDubois2018] Dubois M, Van den Broeck L, Inzé D (2018) The pivotal role of ethylene in plant growth. *Trends Plant Sci* 23: 311–323 29428350 10.1016/j.tplants.2018.01.003 PMC5890734

[REhinmitan2024] Ehinmitan E, Losenge T, Mamati E, Ngumi V, Juma P, Siamalube B (2024) BioSolutions for green agriculture: Unveiling the diverse roles of plant growth-promoting rhizobacteria. *Int J Microbiol* 2024: 6181491 39238543 10.1155/2024/6181491 PMC11377119

[RFall1996] Fall R, Benson AA (1996) Leaf methanol: The simplest natural product from plants. *Trends Plant Sci* 1: 296–301

[RFleischman1998] Fleischman D, Kramer D (1998) Photsynthetic rhizobia. *Biochim Biophys Acta Bioenerg* 1364: 17–36 10.1016/s0005-2728(98)00011-5 9554937

[RFrenkel1998] Frenkel C, Peters JS, Tieman DM, Tiznado ME, Handa AK (1998) Pectin methylesterase regulates methanol and ethanol accumulation in ripening tomato (*Lycopersicon esculentum*) fruit. *J Biol Chem* 273: 4293–4295 9468474 10.1074/jbc.273.8.4293

[RGlick2014] Glick BR (2014) Bacteria with ACC deaminase can promote plant growth and help to feed the world. *Microbiol Res* 169: 30–39 24095256 10.1016/j.micres.2013.09.009

[RGourion2008] Gourion B, Francez-Charlot A, Vorholt JA (2008) PhyR is involved in the general stress response of *Methylobacterium extorquens* AM1. *J Bacteriol* 190: 1027–1035 18024517 10.1128/JB.01483-07 PMC2223570

[RGourion2006] Gourion B, Rossignol M, Vorholt JA (2006) A proteomic study of *Methylobacterium extorquens* reveals a response regulator essential for epiphytic growth. *Proc Natl Acad Sci USA* 103: 13186–13191 16926146 10.1073/pnas.0603530103 PMC1559774

[RGreen2018] Green PN, Ardley JK (2018) Review of the genus *Methylobacterium* and closely related organisms: A proposal that some *Methylobacterium* species be reclassified into a new genus, *Methylorubrum* gen. nov. *Int J Syst Evol Microbiol* 68: 2727–2748 30024371 10.1099/ijsem.0.002856

[RGrossi2020] Grossi CEM, Fantino E, Serral F, Zawoznik MS, Fernandez Do Porto DA, Ulloa RM (2020) *Methylobacterium* sp. 2A is a plant growth-promoting rhizobacteria that has the potential to improve potato crop yield under adverse conditions. *Front Plant Sci* 11: 71 32127795 10.3389/fpls.2020.00071 PMC7038796

[RGrossi2024] Grossi CEM, Tani A, Mori IC, Matsuura T, Ulloa RM (2024) Plant growth-promoting abilities of *Methylobacterium* sp. 2A involve auxin-mediated regulation of the root architecture. *Plant Cell Environ* 47: 5343–5357 39189962 10.1111/pce.15116

[RHabben2014] Habben JE, Bao X, Bate NJ, DeBruin JL, Dolan D, Hasegawa D, Helentjaris TG, Lafitte RH, Lovan N, Mo H, et al. (2014) Transgenic alteration of ethylene biosynthesis increases grain yield in maize under field drought-stress conditions. *Plant Biotechnol J* 12: 685–693 24618117 10.1111/pbi.12172

[RHari2010] Hari P, Boruah D, Chauhan PS, Yim WJ, Han GH, Sa TM (2010) Comparison of plant growth promoting *Methylobacterium* spp. and exogenous indole-3-acetic acid application on red pepper and tomato seedling development. *Korean J Soil Sci Fert* 43: 96–104

[RHibi2011] Hibi Y, Asai K, Arafuka H, Hamajima M, Iwama T, Kawai K (2011) Molecular structure of La^3+^-induced methanol dehydrogenase-like protein in *Methylobacterium radiotolerans.* *J Biosci Bioeng* 111: 547–549 21256798 10.1016/j.jbiosc.2010.12.017

[d67e2126] Hördt A, López MG, Meier-Kolthoff JP, Schleuning M, Weinhold LM, Tindall BJ, Gronow S, Kyrpides NC, Woyke T, Göker M (2020) Analysis of 1,000+ type-strain genomes substantially improves taxonomic classification of Alphaproteobacteria. *Front Microbiol* 11: 468 32373076 10.3389/fmicb.2020.00468 PMC7179689

[RHornschuh2002] Hornschuh M, Grotha R, Kutschera U (2002) Epiphytic bacteria associated with the bryophyte *Funaria hygrometrica*: Effects of *Methylobacterium* strains on protonema development. *Plant Biol (Stuttg)* 4: 682–687

[RIguchi2018] Iguchi H, Yoshida Y, Fujisawa K, Taga H, Yurimoto H, Oyama T, Sakai Y (2018) KaiC family proteins integratively control temperature-dependent UV resistance in *Methylobacterium extorquens* AM1. *Environ Microbiol Rep* 10: 634–643 29901260 10.1111/1758-2229.12662

[RIqbal2017] Iqbal N, Khan NA, Ferrante A, Trivellini A, Francini A, Khan MIR (2017) Ethylene role in plant growth, development and senescence: Interaction with other phytohormones. *Front Plant Sci* 8: 475 28421102 10.3389/fpls.2017.00475 PMC5378820

[RIvanova2001] Ivanova EG, Doronina NV, Trotsenko IA (2001) Aérobnye metilobakterii sinteziruiut auksiny [Aerobic methylobacteria are capable of synthesizing auxins]. *Mikrobiologiya* 70: 452–458 11558269

[RJaftha2002] Jaftha JB, Strijdom BW, Steyn PL (2002) Characterization of pigmented methylotrophic bacteria which nodulate *Lotononis bainesii.* *Syst Appl Microbiol* 25: 440–449 12421082 10.1078/0723-2020-00124

[RJardine2024] Jardine KJ, Gallo L, Roth M, Upadhyaya S, Northen T, Kosina S, Tcherkez G, Eudes A, Domigues T, Greule M, et al. (2024) The ‘photosynthetic C1 pathway’ links carbon assimilation and growth in California poplar. *Commun Biol* 7: 1469 39516667 10.1038/s42003-024-07142-0 PMC11549359

[RJoe2014] Joe MM, Saravanan VS, Islam MR, Sa T (2014) Development of alginate-based aggregate inoculants of *Methylobacterium* sp. and *Azospirillum brasilense* tested under *in vitro* conditions to promote plant growth. *J Appl Microbiol* 116: 408–423 24188110 10.1111/jam.12384

[RJourand2004] Jourand P, Giraud E, Béna G, Sy A, Willems A, Gillis M, Dreyfus B, de Lajudie P (2004) *Methylobacterium nodulans* sp. nov., for a group of aerobic, facultatively methylotrophic, legume root-nodule-forming and nitrogen-fixing bacteria. *Int J Syst Evol Microbiol* 54: 2269–2273 15545469 10.1099/ijs.0.02902-0

[RJourand2005] Jourand P, Renier A, Rapior S, Miana de Faria S, Prin Y, Galiana A, Giraud E, Dreyfus B (2005) Role of methylotrophy during symbiosis between *Methylobacterium nodulans* and *Crotalaria podocarpa.* *Mol Plant Microbe Interact* 18: 1061–1068 16255245 10.1094/MPMI-18-1061

[RJuma2022] Juma PO, Fujitani Y, Alessa O, Oyama T, Yurimoto H, Sakai Y, Tani A (2022) Siderophore for lanthanide and iron uptake for methylotrophy and plant growth promotion in *Methylobacterium aquaticum* strain 22A. *Front Microbiol* 13: 921635 35875576 10.3389/fmicb.2022.921635 PMC9301485

[RKanukollu2022] Kanukollu S, Remus R, Rücker AM, Buchen-Tschiskale C, Hoffmann M, Kolb S (2022) Methanol utilizers of the rhizosphere and phyllosphere of a common grass and forb host species. *Environ Microbiome* 17: 35 35794633 10.1186/s40793-022-00428-y PMC9258066

[RKawaguchi2011] Kawaguchi K, Yurimoto H, Oku M, Sakai Y (2011) Yeast methylotrophy and autophagy in a methanol-oscillating environment on growing *Arabidopsis thaliana* leaves. *PLoS One* 6: e25257 21966472 10.1371/journal.pone.0025257 PMC3180373

[RKeltjens2014] Keltjens JT, Pol A, Reimann J, Op den Camp HJ (2014) PQQ-dependent methanol dehydrogenases: Rare-earth elements make a difference. *Appl Microbiol Biotechnol* 98: 6163–6183 24816778 10.1007/s00253-014-5766-8

[RKhan2024] Khan S, Alvi AF, Saify S, Iqbal N, Khan NA (2024) The ethylene biosynthetic enzymes, 1-aminocyclopropane-1-carboxylate (ACC) synthase (ACS) and ACC oxidase (ACO): The less explored players in abiotic stress tolerance. *Biomolecules* 14: 90 38254690 10.3390/biom14010090 PMC10813531

[RKnief2012] Knief C, Delmotte N, Chaffron S, Stark M, Innerebner G, Wassmann R, von Mering C, Vorholt JA (2012) Metaproteogenomic analysis of microbial communities in the phyllosphere and rhizosphere of rice. *ISME J* 6: 1378–1390 22189496 10.1038/ismej.2011.192 PMC3379629

[RKoenig2002] Koenig RL, Morris RO, Polacco JC (2002) tRNA is the source of low-level trans-zeatin production in *Methylobacterium* spp. *J Bacteriol* 184: 1832–1842 11889088 10.1128/JB.184.7.1832-1842.2002 PMC134930

[d67e2458] Koskimäki JJ, Pirttilä AM, Ihantola EL, Halonen O, Frank AC (2015) The intracellular Scots pine shoot symbiont *Methylobacterium extorquens* DSM13060 aggregates around the host nucleus and encodes eukaryote-like proteins. *mBio* 6: e00039-15 25805725 10.1128/mBio.00039-15 PMC4453540

[RKrug2020] Krug L, Morauf C, Donat C, Müller H, Cernava T, Berg G (2020) Plant growth-promoting methylobacteria selectively increase the biomass of biotechnologically relevant microalgae. *Front Microbiol* 11: 427 32256478 10.3389/fmicb.2020.00427 PMC7093331

[RKumar2021] Kumar M, Giri VP, Pandey S, Gupta A, Patel MK, Bajpai AB, Jenkins S, Siddique KHM (2021) Plant-growth-promoting rhizobacteria emerging as an effective bioinoculant to improve the growth, production, and stress tolerance of vegetable crops. *Int J Mol Sci* 22: 12245 34830124 10.3390/ijms222212245 PMC8622033

[RKwak2014] Kwak MJ, Jeong H, Madhaiyan M, Lee Y, Sa TM, Oh TK, Kim JF (2014) Genome information of *Methylobacterium oryzae*, a plant-probiotic methylotroph in the phyllosphere. *PLoS One* 9: e106704 25211235 10.1371/journal.pone.0106704 PMC4161386

[RLeducq2022] Leducq JB, Sneddon D, Santos M, Condrain-Morel D, Bourret G, Martinez-Gomez NC, Lee JA, Foster JA, Stolyar S, Shapiro BJ, et al. (2022) Comprehensive phylogenomics of *Methylobacterium* reveals four evolutionary distinct groups and underappreciated phyllosphere diversity. *Genome Biol Evol* 14: evac123 35906926 10.1093/gbe/evac123 PMC9364378

[RLindow2003] Lindow SE, Brandl MT (2003) Microbiology of the phyllosphere. *Appl Environ Microbiol* 69: 1875–1883 12676659 10.1128/AEM.69.4.1875-1883.2003 PMC154815

[d67e2564] Ludwig-Müller J (2015) Bacteria and fungi controlling plant growth by manipulating auxin: Balance between development and defense. *J Plant Physiol* 172: 4–12 25456606 10.1016/j.jplph.2014.01.002

[RMadhaiyan2015] Madhaiyan M, Alex TH, Ngoh ST, Prithiviraj B, Ji L (2015) Leaf-residing *Methylobacterium* species fix nitrogen and promote biomass and seed production in *Jatropha curcas.* *Biotechnol Biofuels* 8: 222 26697111 10.1186/s13068-015-0404-y PMC4687150

[RMadhaiyan2009] Madhaiyan M, Poonguzhali S, Kwon SW, Sa TM (2009) *Methylobacterium phyllosphaerae* sp. nov., a pink-pigmented, facultative methylotroph from the phyllosphere of rice. *Int J Syst Evol Microbiol* 59: 22–27 19126717 10.1099/ijs.0.001693-0

[RMadhaiyan2006] Madhaiyan M, Poonguzhali S, Ryu J, Sa T (2006) Regulation of ethylene levels in canola (*Brassica campestris*) by 1-aminocyclopropane-1-carboxylate deaminase-containing *Methylobacterium fujisawaense.* *Planta* 224: 268–278 16416316 10.1007/s00425-005-0211-y

[RMadhaiyan2007] Madhaiyan M, Poonguzhali S, Sa T (2007) Characterization of 1-aminocyclopropane-1-carboxylate (ACC) deaminase containing *Methylobacterium oryzae* and interactions with auxins and ACC regulation of ethylene in canola (*Brassica campestris*). *Planta* 226: 867–876 17541630 10.1007/s00425-007-0532-0

[RMadhaiyan2004] Madhaiyan M, Poonguzhali S, Senthilkumar M, Seshadri S, Chung H, Yang J, Sundaram S, Tongmin S (2004) Growth promotion and induction of systemic resistance in rice cultivar Co-47 (*Oryza sativa* L.) by *Methylobacterium* spp. *Bot Bull Acad Sin* 45: 315–324

[RMizuno2012] Mizuno M, Yurimoto H, Yoshida N, Iguchi H, Sakai Y (2012) Distribution of pink-pigmented facultative methylotrophs on leaves of vegetables. *Biosci Biotechnol Biochem* 76: 578–580 22451403 10.1271/bbb.110737

[RMuday2012] Muday GK, Rahman A, Binder BM (2012) Auxin and ethylene: Collaborators or competitors? *Trends Plant Sci* 17: 181–195 22406007 10.1016/j.tplants.2012.02.001

[d67e2734] Müller DB, Vogel C, Bai Y, Vorholt JA (2016) The plant microbiota: Systems-level insights and perspectives. *Annu Rev Genet* 50: 211–234 27648643 10.1146/annurev-genet-120215-034952

[RNemecek-Marshall1995] Nemecek-Marshall M, MacDonald RC, Franzen JJ, Wojciechowski CL, Fall R (1995) Methanol emission from leaves (enzymatic detection of gas-phase methanol and relation of methanol fluxes to stomatal conductance and leaf development). *Plant Physiol* 108: 1359–1368 12228547 10.1104/pp.108.4.1359 PMC157513

[ROchsner2015] Ochsner AM, Sonntag F, Buchhaupt M, Schrader J, Vorholt JA (2015) *Methylobacterium extorquens*: Methylotrophy and biotechnological applications. *Appl Microbiol Biotechnol* 99: 517–534 25432674 10.1007/s00253-014-6240-3

[ROeum2024] Oeum K, Suong M, Uon K, Jobert L, Bellafiore S, Comte A, Thomas E, Kuok F, Moulin L (2024) Comparison of plant microbiota in diseased and healthy rice reveals methylobacteria as health signatures with biocontrol capabilities. *Front Plant Sci* 15: 1468192 39534110 10.3389/fpls.2024.1468192 PMC11554501

[ROmer2004] Omer ZS, Tombolini R, Gerhardson B (2004) Plant colonization by pink-pigmented facultative methylotrophic bacteria (PPFMs). *FEMS Microbiol Ecol* 47: 319–326 19712320 10.1016/S0168-6496(04)00003-0

[RPalberg2022] Palberg D, Kisiała A, Jorge GL, Emery RJN (2022) A survey of *Methylobacterium* species and strains reveals widespread production and varying profiles of cytokinin phytohormones. *BMC Microbiol* 22: 49 35135483 10.1186/s12866-022-02454-9 PMC8822675

[RPascual2020] Pascual JA, Ros M, Martínez J, Carmona F, Bernabé A, Torres R, Lucena T, Aznar R, Arahal DR, Fernández F (2020) *Methylobacterium symbioticum* sp. nov., a new species isolated from spores of *Glomus iranicum* var. *tenuihypharum*. *Curr Microbiol* 77: 2031–2041 32594222 10.1007/s00284-020-02101-4

[RPatt1976] Patt TE, Cole GC, Hanson RS (1976) *Methylobacterium*, a new genus of facultatively methylotrophic bacteria. *Int J Syst Bacteriol* 26: 226–229

[RPatten2013] Patten CL, Blakney AJ, Coulson TJ (2013) Activity, distribution and function of indole-3-acetic acid biosynthetic pathways in bacteria. *Crit Rev Microbiol* 39: 395–415 22978761 10.3109/1040841X.2012.716819

[RPingali2012] Pingali PL (2012) Green revolution: Impacts, limits, and the path ahead. *Proc Natl Acad Sci USA* 109: 12302–12308 22826253 10.1073/pnas.0912953109 PMC3411969

[RPohjanen2014] Pohjanen J, Koskimäki JJ, Sutela S, Ardanov P, Suorsa M, Niemi K, Sarjala T, Häggman H, Pirttilä AM (2014) Interaction with ectomycorrhizal fungi and endophytic *Methylobacterium* affects nutrient uptake and growth of pine seedlings in vitro. *Tree Physiol* 34: 993–1005 25149086 10.1093/treephys/tpu062

[RPol2014] Pol A, Barends TR, Dietl A, Khadem AF, Eygensteyn J, Jetten MS, Op den Camp HJ (2014) Rare earth metals are essential for methanotrophic life in volcanic mudpots. *Environ Microbiol* 16: 255–264 24034209 10.1111/1462-2920.12249

[RPoonguzhali2008] Poonguzhali S, Madhaiyan M, Yim WJ, Kim KA, Sa TM (2008) Colonization pattern of plant root and leaf surfaces visualized by use of green-fluorescent-marked strain of *Methylobacterium suomiense* and its persistence in rhizosphere. *Appl Microbiol Biotechnol* 78: 1033–1043 18320187 10.1007/s00253-008-1398-1

[RPoorniammal2009] Poorniammal R, Sundaram SP, Kumutha K (2009) In vitro biocontrol activity of *Methylobacterium extorquens* against fungal pathogens. *Int J Plant Prot* 2: 59–62

[RPrasad2022] Prasad R (2022) Cytokinin and its key role to enrich the plant nutrients and growth under adverse conditions-an update. *Front Genet* 13: 883924 35795201 10.3389/fgene.2022.883924 PMC9252289

[RQin2019] Qin H, He L, Huang R (2019) The coordination of ethylene and other hormones in primary root development. *Front Plant Sci* 10: 874 31354757 10.3389/fpls.2019.00874 PMC6635467

[RRaja2006] Raja P, Uma S, Sundaram S (2006) Non-nodulating pink-pigmented facultative *Methylobacterium* sp. with a functional *nifH* gene. *World J Microbiol Biotechnol* 22: 1381–1384

[RRodrigues2024] Rodrigues MÂ, Correia CM, Arrobas M (2024) The application of a foliar spray containing *Methylobacterium symbioticum* had a limited effect on crop yield and nitrogen recovery in field and pot-grown maize. *Plants* 13: 2909 39458855 10.3390/plants13202909 PMC11510831

[RRossetto2011] Rossetto PB, Dourado MN, Quecine MC, Andreote FD, Araújo WL, Azevedo JL, Pizzirani-Kleiner AA (2011) Specific plant induced biofilm formation in *Methylobacterium* species. *Braz J Microbiol* 42: 878–883 24031703 10.1590/S1517-83822011000300006 PMC3768801

[d67e3109] Růžička K, Ljung K, Vanneste S, Podhorská R, Beeckman T, Friml J, Benková E (2007) Ethylene regulates root growth through effects on auxin biosynthesis and transport-dependent auxin distribution. *Plant Cell* 19: 2197–2212 17630274 10.1105/tpc.107.052126 PMC1955700

[RRyback2022] Ryback B, Bortfeld-Miller M, Vorholt JA (2022) Metabolic adaptation to vitamin auxotrophy by leaf-associated bacteria. *ISME J* 16: 2712–2724 35987782 10.1038/s41396-022-01303-x PMC9666465

[RRyu2006] Ryu J, Madhaiyan M, Poonguzhali S, Yim W, Indiragandhi P, Kim K, Anandham R, Yun J, Kim K, Sa T (2006) Plant growth substances produced by *Methylobacterium* spp. and their effect on tomato (*Lycopersicon esculentum* L.) and red pepper (*Capsicum annuum* L.) growth. *J Microbiol Biotechnol* 16: 1622–1628

[RSahin2008] Sahin N, Kato Y, Yilmaz F (2008) Taxonomy of oxalotrophic *Methylobacterium* strains. *Naturwissenschaften* 95: 931–938 18581089 10.1007/s00114-008-0405-9

[RSchauer2011] Schauer S, Kutschera U (2011) A novel growth-promoting microbe, *Methylobacterium funariae* sp. nov., isolated from the leaf surface of a common moss. *Plant Signal Behav* 6: 510–515 21673511 10.4161/psb.6.4.14335 PMC3142378

[RSchmidt2010] Schmidt S, Christen P, Kiefer P, Vorholt JA (2010) Functional investigation of methanol dehydrogenase-like protein XoxF in *Methylobacterium extorquens* AM1. *Microbiology (Reading)* 156: 2575–2586 20447995 10.1099/mic.0.038570-0

[RSenthilkumar2009] Senthilkumar M, Madhaiyan M, Sundaram S, Kannaiyan S (2009) Intercellular colonization and growth promoting effects of *Methylobacterium* sp. with plant-growth regulators on rice (*Oryza sativa* L. Cv CO-43). *Microbiol Res* 164: 92–104 17207982 10.1016/j.micres.2006.10.007

[RShi2017] Shi J, Gao H, Wang H, Lafitte HR, Archibald RL, Yang M, Hakimi SM, Mo H, Habben JE (2017) ARGOS8 variants generated by CRISPR-Cas9 improve maize grain yield under field drought stress conditions. *Plant Biotechnol J* 15: 207–216 27442592 10.1111/pbi.12603 PMC5258859

[RSkovran2019] Skovran E, Raghuraman C, Martinez-Gomez NC (2019) Lanthanides in methylotrophy. *Curr Issues Mol Biol* 33: 101–116 31166187 10.21775/cimb.033.101

[RStreet2015] Street IH, Aman S, Zubo Y, Ramzan A, Wang X, Shakeel SN, Kieber JJ, Schaller GE (2015) Ethylene inhibits cell proliferation of the *Arabidopsis* root meristem. *Plant Physiol* 169: 338–350 26149574 10.1104/pp.15.00415 PMC4577392

[RSun2021] Sun W, Shahrajabian MH, Cheng QI (2021) Nitrogen fixation and diazotrophs: A review. *Rom Biotechnol Lett* 26: 2834–2845

[RSy2001] Sy A, Giraud E, Jourand P, Garcia N, Willems A, de Lajudie P, Prin Y, Neyra M, Gillis M, Boivin-Masson C, et al. (2001) Methylotrophic *Methylobacterium* bacteria nodulate and fix nitrogen in symbiosis with legumes. *J Bacteriol* 183: 214–220 11114919 10.1128/JB.183.1.214-220.2001 PMC94868

[RSy2005] Sy A, Timmers AC, Knief C, Vorholt JA (2005) Methylotrophic metabolism is advantageous for *Methylobacterium extorquens* during colonization of *Medicago truncatula* under competitive conditions. *Appl Environ Microbiol* 71: 7245–7252 16269765 10.1128/AEM.71.11.7245-7252.2005 PMC1287603

[RTang2023] Tang J, Li Y, Zhang L, Mu J, Jiang Y, Fu H, Zhang Y, Cui H, Yu X, Ye Z (2023) Biosynthetic pathways and functions of indole-3-acetic acid in microorganisms. *Microorganisms* 11: 2077 37630637 10.3390/microorganisms11082077 PMC10459833

[RTani2023] Tani A, Masuda S, Fujitani Y, Iga T, Haruna Y, Kikuchi S, Shuaile W, Lv H, Katayama S, Yurimoto H, et al. (2023) Metabolism-linked methylotaxis sensors responsible for plant colonization in *Methylobacterium aquaticum* strain 22A. *Front Microbiol* 14: 1258452 37901831 10.3389/fmicb.2023.1258452 PMC10613068

[RTani2015] Tani A, Sahin N, Fujitani Y, Kato A, Sato K, Kimbara K (2015) *Methylobacterium* species promoting rice and barley growth and interaction specificity revealed with whole-cell matrix-assisted laser desorption/ionization-time-of-flight mass spectrometry (MALDI-TOF/MS) analysis. *PLoS One* 10: e0129509 26053875 10.1371/journal.pone.0129509 PMC4460032

[RTani2012] Tani A, Sahin N, Matsuyama Y, Enomoto T, Nishimura N, Yokota A, Kimbara K (2012) High-throughput identification and screening of novel *Methylobacterium* species using whole-cell MALDI-TOF/MS analysis. *PLoS One* 7: e40784 22808262 10.1371/journal.pone.0040784 PMC3395638

[RTorresVera2024] Torres Vera R, Bernabé García AJ, Carmona Álvarez FJ, Martínez Ruiz J, Fernández Martín F (2024) Application and effectiveness of *Methylobacterium symbioticum* as a biological inoculant in maize and strawberry crops. *Folia Microbiol (Praha)* 69: 121–131 37526803 10.1007/s12223-023-01078-4 PMC10876812

[RUrakami1984] Urakami T, Komagata K (1984) Protomonas, a new genus of facultatively methylotrophic bacteria. *Int J Syst Bacteriol* 34: 188–201

[RValente2024] Valente F, Panozzo A, Bozzolin F, Barion G, Bolla PK, Bertin V, Potestio S, Visioli G, Wang Y, Vamerali T (2024) Growth, photosynthesis and yield responses of common wheat to foliar application of *Methylobacterium symbioticum* under decreasing chemical nitrogen fertilization. *Agriculture* 14: 1670

[RVerginer2010] Verginer M, Siegmund B, Cardinale M, Müller H, Choi Y, Míguez CB, Leitner E, Berg G (2010) Monitoring the plant epiphyte *Methylobacterium extorquens* DSM 21961 by real-time PCR and its influence on the strawberry flavor. *FEMS Microbiol Ecol* 74: 136–145 20662926 10.1111/j.1574-6941.2010.00942.x

[RVorholt2012] Vorholt JA (2012) Microbial life in the phyllosphere. *Nat Rev Microbiol* 10: 828–840 23154261 10.1038/nrmicro2910

[RVu2016] Vu HN, Subuyuj GA, Vijayakumar S, Good NM, Martinez-Gomez NC, Skovran E (2016) Lanthanide-dependent regulation of methanol oxidation systems in *Methylobacterium extorquens* AM1 and their contribution to methanol growth. *J Bacteriol* 198: 1250–1259 26833413 10.1128/JB.00937-15 PMC4859578

[RWormit2018] Wormit A, Usadel B (2018) The multifaceted role of pectin methylesterase inhibitors (PMEIs). *Int J Mol Sci* 19: 2878 30248977 10.3390/ijms19102878 PMC6213510

[RYim2013] Yim W, Seshadri S, Kim K, Lee G, Sa T (2013) Ethylene emission and PR protein synthesis in ACC deaminase producing *Methylobacterium* spp. inoculated tomato plants (*Lycopersicon esculentum* Mill.) challenged with *Ralstonia solanacearum* under greenhouse conditions. *Plant Physiol Biochem* 67: 95–104 23558008 10.1016/j.plaphy.2013.03.002

[RYoshida2017] Yoshida S, Hiradate S, Koitabashi M, Kamo T, Tsushima S (2017) Phyllosphere *Methylobacterium* bacteria contain UVA-absorbing compounds. *J Photochem Photobiol B* 167: 168–175 28068611 10.1016/j.jphotobiol.2016.12.019

[RYoshida2019] Yoshida Y, Iguchi H, Sakai Y, Yurimoto H (2019) Pantothenate auxotrophy of *Methylobacterium* spp. isolated from living plants. *Biosci Biotechnol Biochem* 83: 569–577 30475153 10.1080/09168451.2018.1549935

[RZhao2023] Zhao Z, Wang L, Khan RAA, Song X, Najeeb S, Zhao J, Yang Y, Ling J, Mao Z, Jiang X, et al. (2023) *Methylorubrum rhodesianum* M520 as a biocontrol agent against *Meloidogyne incognita* (Tylenchida: Heteroderidae) J2s infecting cucumber roots. *J Appl Microbiol* 134: lxad001 36611228 10.1093/jambio/lxad001

